# Supplementary material for: The impact of background liver disease on the long-term prognosis of very-early-stage HCC after ablation therapy
Source: PLoS One. 2022 Feb 23;17(2):e0264075. doi: 10.1371/journal.pone.0264075 (PMC8865683; doi:10.1371/journal.pone.0264075)
Supplement: Attachment — Submitted filename: Response to Reviewers.docx [file pone.0264075.s001.docx]

Dear Editor

Thank you very much for your kind review of our manuscript. Taking into account all the suggestions and criticism raised by the editors and reviewers, we have now revised our manuscript. The changes made in the text are highlighted by bold text with an underline. A point-by-point reply to the reviewers’ comments is attached. We believe that we have successfully answered all the suggestions and criticisms and that our manuscript is now suitable for publication. We look forward to hearing from you.

Sincerely,

**Point-by-points to reviewer 1**

#1. Are there any exclusion criteria?

**Reply:** Consecutive patients treated with RFA were included in the present study. Therefore, there were no exclusion criteria for this study. This information is now described in the Methods section.

#2. The authors should describe the detail of the RFA procedure, such as approach, device, or protocol.

**Reply:** Thank you for your suggestion. We have now described the detail of the RFA procedure in the Methods section as follows:

All RFA procedures were performed percutaneously under Local anesthesia and transvenous analgesic administration. Real-time ultrasound (US) was chosen as the guidance modality. We used artificial ascites for tumors, which were in the hepatic dome or adjacent to the gastrointestinal tract. When performing RFA, we use a guiding needle with an external insulated sheath and one of the following devices: VIVA^TM^ RF system (STARmed®); Cool-tip^TM^ RFA system (Covidien®). The device was chosen based on the operator’s decision. Ultrasonography was performed with a 3.0- to 6.0-MHz convex probe and the Aloka SSD-5500 (Aloka, Tokyo, Japan), Sonoline Elegra (Siemens, Erlangen, Germany), Aplio XV, XG and 500 systems (Toshiba Medical Systems-Canon Medical Systems, Tokyo, Japan).

#3. The ethics approval number should be clearly stated.

**Reply:** The Ethics Committee of the Musashino Red Cross Hospital approved this study and the ethical approval number: 636, is now described in the Methods section.

#4. I could not find anything about informed consent.

**Reply:** Thank you for pointing out the important point. The information about informed consent is now described in the Methods section as follows:

Written informed consent was obtained from all patients before the RFA procedure. The requirement for written informed consent to be included in the study was waived because of the retrospective design of the study. The privacy of all patients was fully protected. Information about this study was made available to the patients (posted on the hospital website), and the research subjects were given the opportunity to opt out.

#5. The authors should describe the success rate, frequency of complications, and degree of safety margin for RFA.

**Reply:** The degree of safety margin for RFA was set at 5mm. In this study, the clinical success rate of the initial RFA procedure was 92.7%. In patients with residual tumor or insufficient safety margin, additional RFA, trans-arterial chemo-embolization (TACE), hepatic resection, or stereotactic radiotherapy was performed after considering the localization of the disease, liver function, and the presence of comorbidities. In patients who received additional RFA, CR was achieved in all patients. Treatment complications were seen in 2.3% and there were no treatment-related deaths. This is now described in the Methods section.

#6. In Table 1, the range of tumor diameter is listed as 8-20 mm. This study focused on very early-stage HCC with a single lesion measuring less than 20 mm.

**Reply:** Thank you for the comment. This was a typo and was corrected to 8-19mm.

#7. Was there any effect of post-treatment on overall survival (OS)?
**Reply:** Comparing the cases before and after 2009 (the year where sorafenib became available), there was no statistically significant difference in OS and RFS of HBV or NBNC patients. Therefore, the effect of post-treatment of the patients seems to be minimal in this study cohort. This is now described in the Results section.

#8. In general, liver function is an important prognostic factor for HCC. The authors should consider that modified albumin-bilirubin was not extracted as an independent factor contributing to OS. In this study, was there a statistically significant difference in the changes in liver function between the HCV SVR group and the HCV non-SVR group?

**Reply:** Thank you for indicating the important point. In the HCV SVR group. The following data on ALBI is now described in the Results section and the Discussions section.

The ALBI scores at the time of treatment, 1, 3, and 5 years were -2.56±0.38, -2.67±0.38, -2.83±0.42, and -2.82±0.51 (p=0.006), respectively, and liver function showed a trend toward improvement. On the other hand, in the HCV non-SVR group, the ALBI scores at the time of treatment, 1, 3, and 5 years were -2.29±0.49, -2.21±0.51, -2.21±0.56, and -2.13±0.59 (p=0.3), respectively, and liver function did not change significantly. Therefore, the improvement in liver function may be one of the reasons for the improved prognosis after treatment in the HCV SVR group.

**Point-by-points to reviewer 2**

#1. The study period for the inclusion of patients is quite long, from 1999 to 2020. Since the advancement of medicine in this long period, OS and RFS may differ between patients who were included earlier and those who were included more recently, and such differences may affect the study results. Therefore, authors better show and/or describe the impact of the inclusion period of the patients.

**Reply:** Thank you for the important comment. Comparing the cases before and after 2011 (the year where DAA became available), there was no statistically significant difference in OS and RFS of HBV or NBNC patients. So, we think that the impact of the inclusion period of the patients was minimal. This is now described in the Results section.

#2. In relation to 1), in the era of IFN therapies, many patients could not achieve SVR. If so, is it possible that most of the HCC patients with active HCV infection participated in the early days of this study?

**Reply:** As you pointed out, among the patients who underwent RFA before 2011, only 6 patients achieved SVR (6.3%). The other patients did not achieve SVR during the observation period.

#3. What is a suspected mechanism for the improvement of survival in HCV-HCC patients with SVR? Did anti-HCV therapy decrease the HCC recurrence rate? Or did anti-HCV therapy have an impact on the maintaining liver functional reserve in HCC therapy? The authors should compare the clinical course of liver function in those with and without SVR.

**Reply:** One of the suspected mechanisms for the improvement of survival in HCV-HCC patients with SVR is the improvement of liver function: The following data is now presented in the Results section and the Discussions section.

In the HCV SVR group, the ALBI scores at the time of treatment, 1, 3, and 5 years were -2.56±0.38, -2.67±0.38, -2.83±0.42, and -2.82±0.51(p=0.006), respectively, and liver function showed a trend toward improvement. On the other hand, in the HCV non-SVR group, the ALBI scores at the time of treatment, 1, 3, and 5 years were -2.29±0.49, -2.21±0.51, -2.21±0.56, and -2.13±0.59(p=0.3), respectively, and liver function did not change significantly. Therefore, the improvement in liver function may be one of the reasons for the improved prognosis after treatment in the HCV SVR group.

#4. In Figures 3 and 4, the OS and RFS of HBV, NBNC, and HCV-SVR also should be separately demonstrated.

**Reply:** Thank you for the suggestions. The figures and corresponding figure legends are corrected as suggested.
